# Essential oil of the leaves of *Ricinus communis* L.: *In vitro* cytotoxicity and antimicrobial properties

**DOI:** 10.1186/1476-511X-11-102

**Published:** 2012-08-13

**Authors:** Zied Zarai, Ines Ben Chobba, Riadh Ben Mansour, Ahmed Békir, Néji Gharsallah, Adel Kadri

**Affiliations:** 1 Laboratoire de Biochimie et de Génie Enzymatique des Lipases, ENIS, BPW, University of Sfax, 1173, Sfax, Tunisia; 2 Laboratoire de Biotechnologies Végétales Appliquées à l’Amélioration des Cultures, Faculté des Sciences de Sfax, University of Sfax, B.P. 11713000, Sfax, Tunisia; 3 Unité de recherche Biotechnologie et pathologies, Institut Supérieur de Biotechnologie de Sfax, University of Sfax, Sfax, Tunisia; 4 Département de Génie des procédés, ISET Sfax, University of Sfax, Km 2,5 Rte de Mahdia, 3099, Sfax, Tunisia

**Keywords:** *R. communis*, Essential oil, Antimicrobial activity, Cytotoxicity

## Abstract

**Background:**

The aim of the present study was to appraise the antimicrobial activity of *Ricinus communis* L. essential oil against different pathogenic microorganisms and the cytotoxic activity against HeLa cell lines.

**Methods:**

The agar disk diffusion method was used to study the antibacterial activity of *Ricinus communis* L. essential oil against 12 bacterial and 4 fungi strains. The disc diameters of zone of inhibition (DD), the minimum inhibitory concentrations (MIC) and the concentration inhibiting 50% (IC_50_) were investigated to characterize the antimicrobial activities of this essential oil. The *in vitro* cytotoxicity of *Ricinus communis* L. essential oil was examined using a modified MTT assay; the viability and the IC_50_ were used to evaluate this test.

**Results:**

The essential oil from the leaves of *Ricinus communis* L. was analyzed by GC–MS and bioassays were carried out. Five constituents of the oil were identified by GC–MS. The antimicrobial activity of the oil was investigated in order to evaluate its efficacy against twelve bacteria and four fungi species, using disc diffusion and minimum inhibitory concentration methods. The essential oil showed strong antimicrobial activity against all microorganisms tested with higher sensitivity for *Bacillus subtilis*, *Staphylococcus aureus* and *Enterobacter cloacae*. The cytotoxic and apoptotic effects of the essential oil on HeLa cell lines were examined by MTT assay. The cytotoxicity of the oil was quite strong with IC50 values less than 2.63 mg/ml for both cell lines.

**Conclusion:**

The present study showed the potential antimicrobial and anticarcinogenic properties of the essential oil of *Ricinus communis* L., indicating the possibilities of its potential use in the formula of natural remedies for the topical treatment of infections.

## Background

Essential oils obtained from aromatic plants have recently gained popularity and scientific interest. Many plants are used for different industrial purposes such as food, drugs, and perfumery manufacturing [1-3]. These compounds possess a wide spectrum of pharmacological activities [4]. They also do not enhance the “antibiotic resistance”, a phenomenon caused by long-term use of synthetic antibiotics. However, due to an increasing use of herbal products, a special care should be given to their safety, effectiveness, and drug interactions.

The dramatic increase of infectious diseases, especially those caused by microbial contamination of foods, has become, particularly in underdeveloped countries, an urgent priority [5-9]. Accordingly, there is a need to develop alternative antimicrobial drugs for their treatment. The use of local medicinal plants for possible antimicrobial and antifungal applications represents a serious promise to satisfy this need. Recently, there has been an increasing interest in essential oils as potential source of natural and safe antioxidants for food industry [5,6,9-11].

*Ricinus communis* (Euphorbiaceae family) is a soft wooden small tree developed throughout tropics and warm temperature regions. Having an antimicrobial activity, this plant was used to cure different ailments. It also inhibits the mitochondrial respiratory chain reactions [12]. Its leaf, root, and seed oil represent a therapeutic potential including inflammation treatment, liver disorders, hypoglycemic, and laxative [13-15]. In Tunisia, *Ricinus communis* is used as a contraceptive herbal drug. In addition, it is a traditional folk medicine used in the treatment of warts, cold tumors, and indurations of mammary glands, corns, and moles [16-18]. Previous studies e.g., [19] proved the anti-inflammatory and the free radical scavenging activity. It was also reported that the essential oil of this plant possesses a moderate antioxidant activity [20]. However, the antimicrobial and cytotoxic properties of *R. communis* essential oil have not yet been explicitly discussed.

In the present study, we investigated the antibacterial and the antifungal activities of *R. communis* essential oil against several pathogenic microorganisms. We also studied its antiproliferative properties against HeLa cell lines.

## Methods

### Chemicals, reagents and plant material

**Figure 1:**
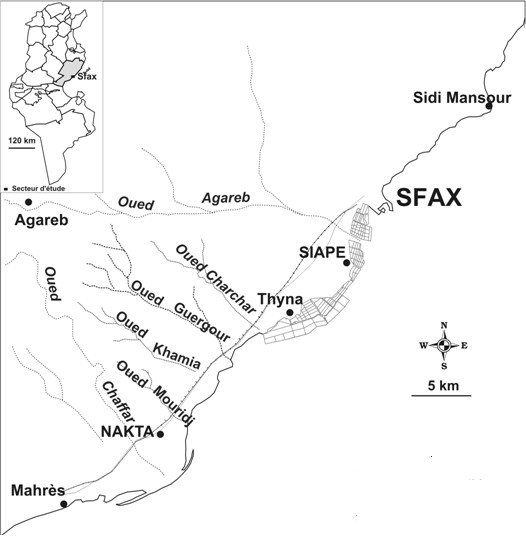
**Geographical localization of the site of Tunisian *R. communis *population (from the region of Guergour, Sfax, south of Tunisia).**

Chemicals and reagents were supported by Prolabo (Paris, France) and Pharmacia (Uppsala, Swedeen). The aerial part (leaves) of *R. communis* was collected during the beginning flowering stage in April 2009 from the region of Guergour, Sfax (south of Tunisia) (Figure 1). The plant materials were confirmed by A. Bekir. Voucher specimens were deposited at ISET, Sfax (Département de Génie des procédés) as Bekir 231.

### Distillation of essential oil and GC/MS analysis conditions

The fresh aerial parts of *R. communis* (300 g) were hydrodistilled using a Clevenger-type apparatus to recover the essential oils for 4 h. The distilled essential oils were dried over anhydrous sodium sulfate, filtered, and stored at +4°C.

The *R. communis* essential oil was analyzed using an Agilent-Technologies 6890N Network GC system equipped with a flame ionization detector and HP-5MS capillary column (30 m × 0.25 mm, film thickness 0.25 μm; Agilent-Technologies, Little Falls, CA, USA). The injector and detector temperatures were set at 250 and 280°C, respectively. The column temperature was programmed from 35 to 250°C at a rate of 5°C/min, with the lower and upper temperatures being held for 3 and 10 min, respectively. The flow rate of the carrier gas (helium) was 1.0 ml/min. A sample of 1.0 μl was injected, using split mode (split ratio, 1:100). All quantifications were carried out using a built-in data-handling program provided by the manufacturer of the gas chromatograph. The composition was reported as a relative percentage of the total peak area. The identification of the essential oil constituents was based on a comparison of their retention times to *n*-alkanes, compared to published data and spectra of authentic compounds. Compounds were further identified and authenticated using their mass spectra compared to the Wiley version 7.0 library.

### Antimicrobial activity assay

#### Microbial strain

The antimicrobial activity of the *R. communis* essential oil was investigated against sixteen of pathogenic microbial strains. The test microorganisms used for antimicrobial sensitivity testing including twelve species of bacteria (*Staphylococcus aureus* 1327, *Staphylococcus epidermidis*, *Micrococcus luteus*, *Enterococcus faecalis*, *Enterobacter cloacae*, *Staphylococcus aureus* 25923, *Bacillus subtilis*, *Bacillus cereus*, *Pseudomonas aeruginosa 27853*, *Klebsiella pneumoniae* WHO24, *Escherchia coli* 25922) and four species of fungi (*Botrytis cinerea*, *Fusarium solani*, *Penicillium digitatum* and *Aspergillus niger*) were used in this study.

#### Agar diffusion method

The agar diffusion method was used for the determination of antibacterial activities of *R. communis* essential oil according to the method described by Berghe and Vlietinck (1991) [21]. Prior to analysis, the essential oil was dissolved in absolute ethanol to create final concentration of 0.1 mg/ml and sterilized by filtration trough 0.22 μm Nylon membrane filter. Different concentrations of the *R. communis* essential oil were used to set a correlation between oil activity and its dose. The bacterial strains were cultured in a nutriment broth for 24 hours. Then, 200 μl of each suspension bacteria (10^6^CFU estimated by absorbance at 600 nm) was spread on Luria Broth agar. Wells were made by using a sterile borer and were loaded with 10 μl of each sample extract. Ampicillin (10 μg/well) was used as positive control. All plates were incubated at 37°C for 24 hours. Antibacterial activity was evaluated by measuring the zone of inhibition in millimetres. All experiments were carried out in triplicates.

#### Determination of the minimal inhibitory concentration (MIC)

The Minimal Inhibitory Concentration (MIC) was obtained by a broth microdilution method [22] testing, which was based on reference method M38-P recommended by the NCCLS [23]. The inoculum of each bacterium was prepared and the suspensions were adjusted to 10^6^CFU/ml. Essential oil was dissolved in absolute ethanol. Then, diluted series were prepared in a 96-well plate. Each well of the microplate included 40 μl of the growth medium, 10 μl of inoculums and 50 μl of the diluted sample of oil. The ampicillin and ethanol were used as positive and negative controls, respectively. Plates were then covered with the sterile plate and incubated at 37°C for 24 h. Subsequently, 40 μl of 3-(4,5-dimethyl-thiazol-2-yl)-2,5-diphenyl-tetrazolium bromide (MTT) at a final concentration 0.5 mg/ml freshly prepared in water was added to each well and incubated for 30 min. The change to red colour indicated that the bacteria are biologically active. The MIC was taken to the well, where no change of colour of MTT was observed. The experiments values were carried out in triplicate.

#### Antibacterial assay disc-diffusion method

All tests were performed in MHB supplemented with ethanol 5% [24,25]. Bacterial strains were cultured overnight in MHB at 37°C. Tubes of MHB containing various concentrations of essential oil were inoculated with 10 μl bacterial inoculums adjusted to 10^6^CFU/ml. They were incubated under shaking conditions (100-120 rpm) at 37°C for 24 h [26,27]. Control tubes without tested samples were simultaneously assayed. The assays were performed in triplicate.

#### Antifungal assay disc-diffusion method

The biological activity against fungi was determined by employing disc agar diffusion method using Sabouraud Dextrose agar [28]. The *R. communis* essential oil was deposited on sterile paper discs (6 mm diameter) which were subsequently placed in the centre of the inoculated Petri dishes. After an incubation period of the 24 h at 30°C, the inhibitory activity was compared to that of commercial cycloheximide at a concentration of 1 mg/ml.

### Cell lines and culture condition

HeLa cells (cervical cancer line, adherent) were used to investigate the cytotoxicity effect of *R. communis* essential oil. This cell line were grown in RPMI 1640 medium (Gibco) supplemented with 10% (v/v) foetal calf serum (FCS) and 2 mM L-glutamin in tissue culture flasks (Nunc). They were passed twice a week and kept at 37°C in a humidified atmosphere of 95% air and 5% CO_2_.

### MTT test

The proliferation rates of HeLa cells after treatment with *R. communis* essential oil were determined by the colorimetric 3-(4,5-dimethylthiazol-2-yl)-2,5-diphenyl tetrazolium bromide (MTT) assay. The yellow compound MTT is reduced by mitochondrial dehydrogenases to the water-insoluble blue compound formazan, depending on the viability of cells.

HeLa cells (4 × 10^4^ in each well) were incubated in 96-well plates for 24 hours in the presence or absence of essential oil. Twenty microlitres MTT solution (Sigma) (5 mg mL^-1^ in PBS) were added to each well. The plate was incubated for 4 h at 37°C in a CO_2_-incubator. One hundred and eighty microlitres of medium was removed from every well without disturbing the cell clusters. A 180 μl methanol/DMSO solution (50:50) was added to each well, and the preparations were thoroughly mixed on a plate shaker with the cell containing formazan crystals. After the dissolution of all crystals, the A570 values were determined [29] with a microplate reader (ELx 800).

## Results and discussion

### Antimicrobial assays

The used bacteria and fungi were selected because they are implicated with skin, oral and intestinal tract of man. The *in-vitro* antibacterial activity of *R. communis* essential oil was evaluated by a paper disc diffusion method against sixteen microorganisms. Their potency was assessed quantitatively by Disc Diameters (DD) of inhibition zone, Minimum Inhibitory Concentrations (MIC), and IC_50_ methods. Essential oils exhibited antimicrobial activity against the tested strains. Results are comparable to the antibiotic Ampicillin, used as a positive control. Results (Table 1) show that essential oil inhibited the growth of bacterial strains. Depending on the susceptibility of the tested bacteria, it produced an inhibition zone varying from 6.2 to 28.4 mm for Gram positive bacteria and from 4.2 to 8.2 mm for Gram negative. Among Gram positive bacteria, highest inhibitory zone was observed against *B. subtilis* (28.4 mm) followed by *S. aureus* (24 mm) and *E. cloacae* (22.6 mm). Among Gram negative, highest inhibitory zone was observed against *P. aeruginosa* (8 mm). The inhibition zone for ampicillin (10 μg/disc), which was used as positive controls for bacteria, ranged from 20 to 26 mm.

**Table 1:** **Antibacterial activity of *R. communis *essential oil, using agar disc diffusion, IC**_**50 **_**and minimal inhibition concentration (MIC)**

**Strains**	**DD**^**a**^	**IC**_**50**_^**b**^	**MIC**^**c**^	**DD**^**d**^
Bacterial strains Gram (+)				
* Bacillus subtilis*	28.4 ± 0.8	480 ± 1	>190.00	26 ± 0.6
* Staphylococcus aureus 1327*	24.0 ± 1.0	290 ± 5	>150.00	20 ± 0.5
* Enterobacter cloacae*	22.6 ± 0.7	390.5 ± 19	>150.00	21 ± 1.4
* Staphylococcus epidermidis*	16.6 ± 0.7	210 ± 5	>120.00	22 ± 0.5
* Enterococcus faecalis*	15.2 ± 1.0	290 ± 10	>180.00	25 ± 1.0
* Staphylococcus aureus* 25923	14.0 ± 1.0	500 ± 20	>150.00	24 ± 0.5
* Micrococcus luteus*	8.0 ± 0.5	230 ± 5	>140.00	20 ± 1.5
* Bacillus cereus*	6.1 ± 0.9	300 ± 10	>130.00	21 ± 1.0
Bacterial strains Gram (-)				
* Pseudomonas aeruginosa 27853*	8.2 ± 0.8	530 ± 10	>270.00	20 ± 1.0
* Klebsiella pneumoniae* WHO24	6.2 ± 0.4	652 ± 8	>320.00	21 ± 0.9
* Escherchia coli* 25922	4.2 ± 0.9	430 ± 5	>240.00	22 ± 0.8
* Salmonella*	4.2 ± 0.6	590 ± 10	>250.00	29 ± 1.0

Results are means of three different experiments.

^a^DD: Disc Diameter of inhibition (halo size) in millimeters, Essentiel.oil 100 μg/disc.

^b^MIC: minimum inhibitory concentration (in micrograms per milliliter).

^c^IC_50_: 50% inhibition concentration (in micrograms per milliliter).

^d^DD: Disc Diameter of inhibition zone of Ampicillin (10 μg/disc), was used as positive controls for bacteria.

For the fungi strains (Table 2), the disc diameter zones of inhibition ranged from 4.2-10.2 mm with a maximal inhibition zone obtained for *P. digitatum.*

**Table 2:** **Antifungal activity of *R. communis *essential oil using agar disc diffusion, IC**_**50 **_**and minimal inhibition concentration methods (MIC)**

**Strains**	**DD**^**a**^	**IC**_**50**_^**b**^	**MIC**^**c**^	**DD**^**d**^
Fungal strains				
* Penicillium digitatum*	10.2 ± 0.2	350 ± 20	>140.00	21 ± 0.9
* Fusarium solani*	08.2 ± 0.5	270 ± 30	>190.00	28 ± 0.6
* Botrytis cinerea*	04.2 ± 0.6	590 ± 10	>250.00	29 ± 1.0
* Aspergillus niger*	NA	NA	NA	30 ± 0.5

Results are means of three different experiments.

^a^DD: Disc Diameter of inhibition (halo size) in millimeters, Essentiel.oil 100 μg/disc.

^b^MIC: minimum inhibitory concentration (in micrograms per milliliter).

^c^IC_50_: 50% inhibition concentration (in micrograms per milliliter).

^d^DD: Cycloheximide (10 μg/disc), was used as positive controls for fungi.

NA: no active.

The MIC and IC_50_ values of *R. communis* essential oil on bacteria ranged from 120 μg/ml to 300 μg/ml, and from 210 μg/ml to 870 μg/ml, respectively. Whereas the MIC and IC_50_ values on fungi ranged from 140 μg/ml to 250 μg/ml and from 350 μg/ml to 590 μg/ml.

**Figure 2:**
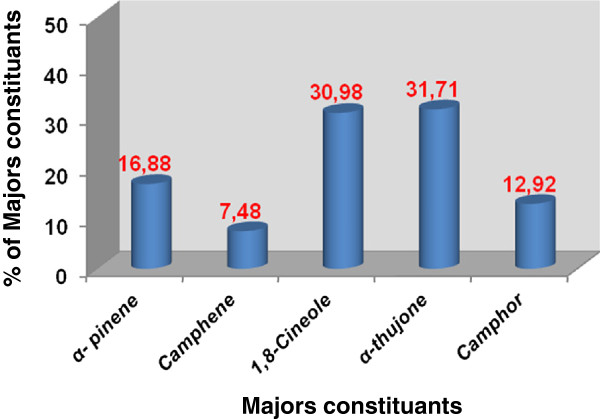
**Chemical composition of *R. communis *essential oil.**

The antimicrobial activity of *R. communis* essential oil is strictly connected to their chemical compositions [30]. As it was reported previously (Figure 2) [20], the GC-MS analysis of *R. communis* essential oil using HP-5MS capillary column led the identification of five compounds accounting for 99.97% of the oil with a yield of 0.32%. The composition was predominantly by α-thujone and 1,8-cineole with equivalent contents of 31.71 and 30.98%, respectively, followed by α-pinene (16.88%), camphor (12.98%) and camphene (7.48%). Our results show that the variation in quantities of the main components *e.g.* camphor and 1,8-cineole, might be responsible for the different antimicrobial activity. Camphor as well as 1,8-cineole was revealed to inhibit the growth of bacteria and fungi [31,32]. Therefore, the detected antimicrobial properties of this essential oil could be due to the relatively high concentration of α-pinene (16.88%), which is believed to actively inhibit the growth of microorganisms [33]. The intense antimicrobial properties of essential oils from the aerial parts of *R. communis* was suspected to be associated with their high contents of oxygenated monoterpene (75.61%) appeared more active against the tested Gram positive than Gram negative bacteria. This result was in agreement with many studies realized on other plant species like *E. robusta*, *E. alba*, *E. camadulensis*, *E. citriodora*, *E. globulus*, *E. saligna*[34]. The effectiveness of this essential oil with containing a camphor at 12.98% against *S. aureus* 1327 (24 mm) and *S. aureus* 25923 (14 mm) are in agreement with those reported in the literature for other essential oil rich in camphor that showed a very strong action versus *S. aureus*[35]. *Artemisia* oils rich in camphor and 1,8-cineole were previously demonstrated to have potent antimicrobial activities *in vitro*[36].

Generally, Gram positive bacteria were more susceptible than Gram negative bacteria. *B. subtilis* and *S. aureus* were the most sensitive. While, *P. aeruginosa, Salmonella and E. coli* were the most resistant strain tested against this essential oil. Our results are in good agreement with the findings of Cantore *et al*. (2004) [37] who reported that Gram positive bacteria are more sensitive to plant essential oils than Gram negative bacteria, especially *E. coli*. The resistance of Gram negative bacteria against essential oils has been attributed to the presence of a hydrophilic outer membrane containing a hydrophilic polysaccharide chain, which acts as a barrier hydrophobic essential oil [38].

Essential oils always represent a complex mixture of different chemical components. Thus, it is very difficult to attribute the antibacterial effect of the total oil to a few active principles. In general, it was also possible that the compounds in lower percentage might be involved in some type of synergism with the active compound [30].

### Cytotoxicity assays

The effect of different concentrations of *R. communis* essential oil on HeLa cell survival was studied. The results summarized in Table 3, show that the *R. communis* essential oil exhibited a moderate inhibitory effect on the cervical cancer line examined. At a concentration of 3 mg/ml, essential oil destructed HeLa cells by about 30%, however at at a concentration of 4 mg/ml, almost all HeLa cells were destructed. Cytotoxicity was expressed as the concentration of oil inhibiting cell growth by 50% (IC_50_). The IC_50_ value of *R. communis* essential oil was evaluated to 2.63 mg/ml. Therefore, doses under this concentration were used for biological antioxidant activity investigation. *R. communis* essential oil was able to exert antiproliferative activity against Hela cell line, this result suggests a specific mechanism of action interfering with abnormal proliferation.

**Table 3:** **Cytotoxic effects of *R. communis *essential oil on HeLa cell line by MTT assay**

**Essential oil (μg/ml)**	**% cell viability**
0	100.00
125	98.90
500	95.77
1000	89.72
1500	84.70
2000	88.80
2200	70.00
2360	60.00
2600	46.00
3000	28.27
3500	01.53
4000	0.210

Results are presented as viability ratio. Values were expressed as the mean of at least three independent experiments.

Our study confirmed the results reported previously by Kadri et al. [20], who indicated that the antioxidant activity of *R. communis* essential oil was efficient when concentrations are less than 0.4 mg/ml. The cytotoxicity of this essential oil was attributed to the presence of α-pinene (16.88%) [39], and to the synergetic effect between minor and major compounds [39].

## Conclusion

In conclusion, essential oil of *R. communis* showed significant antimicrobial and antiproliferative activities. α-thujone, 1,8-cineole, α-pinene, Camphor and camphene were common in the oil as five major compounds. The results suggest that *R. communis* essential oils possess some compounds with antimicrobial and antiproliferative properties, which can be used as antimicrobial agents in new drugs for treatment of infectious diseases. It is quite difficult to attribute the antimicrobial and the cytotoxic effects of an essential oil to one or a few active principles, because extracts always contain a mixture of different chemical compounds. In addition to the major components, also minor components may make a significant contribution to the biological activity of extracts. Following the results above, we could infer that the antimicrobial and the cytotoxic effects of *R. communis* essential oil is the synergistic effect of their compositions.

## Competing interests

The authors declare that they have no competing interests.

## Authors’ contributions

ZZ, IBC, RBM and AB carried out the experimental part such as extraction, antibacterial, antifungal and cytotoxicity assays. ZZ contribute to the analysis of the results. NG and AK supervised the work and corrected the manuscript. Authors read and approved the final manuscript.
